# Robocast Zn- and Co-doped bioactive glass/tricalcium phosphate scaffolds for bone regeneration

**DOI:** 10.1186/s13036-025-00585-x

**Published:** 2025-12-06

**Authors:** Mahdieh Hajian Monfared, Sanam Mohandesnezhad, Mahmoud Azami, Saeed Samani

**Affiliations:** 1 Tissue Engineering and Applied Cell Sciences Division, Department of Anatomical Sciences, Faculty of Medical Sciences, Tarbiat Modares University, Tehran, Iran; 2 Department of Tissue Engineering, School of Advanced Technologies in Medicine, Tehran University of Medical Sciences, Tehran, Iran; 3 Department of Biomedical Engineering, Faculty of Engineering and Natural Sciences, Istinye University, Istanbul, 34396 Turkey; 4 Regenerative Medicine Group (REMED), Universal Scientific Education and Research Network (USERN), Tehran, Iran; 5 Joint Reconstruction Research Center (JRRC), Tehran University of Medical Sciences, Tehran, Iran

**Keywords:** BG/TCP scaffold, hADMSCs, 3D-printing, Bone tissue engineering

## Abstract

**Background:**

Bone tissue engineering requires scaffolds that synergize mechanical strength with bioactivity. This study aimed to develop and characterize 3D-printed zinc (Zn)- and cobalt (Co)-doped 45S5 bioactive glass (BG)/β-tricalcium phosphate (TCP) composite scaffolds for enhanced bone regeneration.

**Methods:**

Sol-gel-synthesized BG powders, doped with 3–15% Zn or 1–5% Co, were combined with TCP (50:50 ratio) to fabricate porous scaffolds via robocasting. Scaffolds were screened for cytocompatibility (MTT assay) and ion release (ICP-OES). The optimal compositions (3% Zn and 1% Co) were characterized for mechanical strength, in vitro bioactivity in SBF, and osteogenic potential using hADMSCs through qPCR, ALP activity, immunocytochemistry, and Alizarin Red staining.

**Result:**

The 3% Zn- and 1% Co-doped scaffolds demonstrated excellent cytocompatibility (≥ 90% hADMSC viability) with controlled ion release. They exhibited compressive strengths of 15.67–20.24 MPa, matching cancellous bone, and significantly accelerated hydroxyapatite formation in SBF. Biologically, the scaffolds induced distinct, stage-specific osteogenic responses. Zn-doping preferentially enhanced early osteogenesis, marked by significantly higher collagen type I (COL-1) expression at day 21 and the highest ALP activity at day 14. In contrast, Co-doping specifically promoted late-stage maturation, resulting in superior osteocalcin (OCN) expression and a 2.5-fold increase in calcium mineralization compared to undoped controls, as quantified by Alizarin Red staining. Immunocytochemistry confirmed this trend, with robust expression of both COL-1 and osteopontin in the doped groups.

**Conclusion:**

The results demonstrate that Zn²⁺ and Co²⁺ ions confer complementary biological functions within BG/TCP scaffolds, effectively promoting sequential stages of osteogenesis. The robocast scaffolds successfully combine mechanical competence with enhanced osteoinductivity, presenting a highly promising platform for the repair of load-bearing bone defects.

## Background

Bone-related diseases affect millions worldwide, making bone grafts the second most common transplant. Given bone’s critical functions, developing solutions for its repair is a major scientific priority [1, 2]. Although autografts are the gold standard, limitations like donor scarcity and risks of disease transmission have increased demand for engineered biomaterials [3–5].

In bone tissue engineering, scaffolds must be biocompatible and support regeneration [6], with material selection and fabrication being critical [7]. Calcium phosphate bioceramics, like hydroxyapatite (HA) and tricalcium phosphate (TCP), are widely used due to their similarity to bone mineral, biocompatibility, and osteoconductivity [8, 9]. However, their brittleness and low mechanical strength remain limitations [10]. Composite biomaterials have thus gained attention for improving properties [11, 12]. Incorporating 45S5 bioactive glass (BG) into calcium phosphate scaffolds, for instance, enhances mechanical performance and bioactivity [5, 13]. The synergy between TCP’s rapid bioresorption and BG’s sustained ion release creates a favorable environment for bone ingrowth and osteoblast proliferation [14, 15].

Bone tissue regeneration is challenging due to the complex interplay of scaffold properties like porosity, mechanics, and biocompatibility, making the process often costly and time-consuming [16, 17]. Advances in 3D printing and computer-aided design (CAD) now allow for optimized scaffold geometry and patient-specific implants via additive manufacturing [18].

Robocasting, or robotic extrusion, has been one of the most widely used direct 3D printing techniques for fabricating dense ceramic structures since its emergence two decades ago [19]. This method involves the continuous layer-by-layer extrusion of filament (ink) through a nozzle, controlled by computer software [20]. Its key advantage over other 3D printing methods lies in the exceptional mechanical strength of the printed structures and its versatility in processing diverse materials, including metallic biomaterials, polymers, bioactive glasses, and ferroelectrics—unlike most 3D printing techniques, which are often limited to specific material types [21, 22]. The robocasting of BG-TCP composites, in particular, has been successfully demonstrated for creating scaffolds with high mechanical integrity and interconnected porosity ideal for bone regeneration [23–25].

A suitable cell source is also crucial in tissue engineering. Adult stem cells are particularly valuable for tissue engineering applications due to their high proliferative potential and minimally invasive harvesting [26]. These cells are undifferentiated, multipotent, and capable of self-renewal, with the ability to differentiate into various mesenchymal lineages, including adipocytes, myocytes, chondrocytes, and osteocytes [27–29]. Human adipose-derived mesenchymal stem cells (hADMSCs) exhibit osteogenic potential comparable to bone marrow-derived MSCs (BMMSCs), with the added advantages of being obtainable through less invasive procedures and demonstrating superior proliferative capacity [30, 31]. However, research indicates that hADMSCs show slightly reduced osteogenic differentiation potential compared to BMMSCs [30]. This limitation can be overcome by using bioactive scaffolds that provide appropriate osteoinductive cues, thereby enhancing the osteogenic commitment of hADMSCs [30, 32]. While current clinical applications remain in experimental stages, hADMSCs represent a promising cell source for future investigations [33].

Numerous inorganic ions including copper, strontium, zinc, cobalt, and cerium are applied in regenerative medicine for their ability to stimulate angiogenesis, osteogenesis, and other therapeutic effects [34], making ion-doping a common strategy for bioactive glasses [35]. Among these, zinc is essential for bone formation and mineralization, enhancing osteogenic activity and alkaline phosphatase at optimal concentrations [36–38]. Similarly, cobalt ions induce hypoxic conditions by triggering HIF-1, promoting bone regeneration, angiogenesis, and fracture repair [39, 40]. These properties make both zinc-doped and cobalt-releasing bioactive glasses promising biomaterials for bone tissue engineering [41–44].

Building directly on our established robocasting platform for robust 45S5 BG/β-TCP scaffolds [23, 45], where BG acts as a bioactive component and sintering aid, this study functionalizes this optimized composite with therapeutic ions. We hypothesized that separately incorporating Zn (to enhance hADMSC osteogenesis) and Co (to promote angiogenesis) would augment biological performance without compromising mechanical advantages. While additive manufacturing of doped bioceramics exists [46–48], a direct comparison of Zn and Co within an identical robocast system is novel. This work thus uniquely combines tunable biochemical stimulation with superior microarchitectural control to create a highly favorable environment for accelerated bone regeneration.

This study presents a systematic investigation of Zn-doped (at 3, 9, 15%) and Co-doped (at 1, 3, 5%) BG/TCP scaffolds fabricated via robocasting, with its novelty rooted in three key contributions: it provides a direct comparison of these specific zinc and cobalt concentrations within an identical robocast scaffold system; it leverages advanced manufacturing to ensure tailored architectures selected through a comprehensive synthesis-to-assessment protocol; and it offers critical biological insights by evaluating performance with human adipose-derived mesenchymal stem cells (hADMSCs). The findings demonstrate that this approach represents a significant advance in developing ion-functionalized bone substitutes.

## Materials and methods

### Materials

Ca(NO₃)₂·4 H₂O, Merck, Germany, diammonium hydrogen phosphate ((NH₄)₂HPO₄, Merck, Germany), tetraethyl orthosilicate (TEOS, C₈H₂₀O₄Si, Merck, Germany), calcium nitrate (Merck, Germany), sodium nitrate (Merck, Germany), triethyl phosphate (TEP, C₆H₁₅O₄P, Sigma-Aldrich), zinc nitrate (Zn(NO₃)₂, Merck, Germany), cobalt nitrate (Co(NO₃)₂, Merck, Germany), Gelatin (Merck, Germany), PVA (Merck, Germany), 3-(4,5-dimethylthiazol-2-yl)-2,5-diphenyltetrazolium bromide (MTT; Sigma-Aldrich), Dulbecco’s modified Eagle’s medium (DMEM high glucose, Gibco, USA), α-MEM supplemented, Fetal bovine serum (FBS; Gibco, USA), dimethyl sulfoxide (DMSO, Merck, Germany), simulated body fluid (SBF, biomaterial, Tehran, Iran), Penicillin/Streptomycin (PBS, Gibco, USA), Hanks medium (Gibco, USA), DMEM F12(Gibco, USA), Trypsin/ EDTA (Gibco, USA), collagenase type (I) (Sigma, Aldrich), phosphate buffer saline (PBS; Gibco, USA), commercial RNA isolation kit (Yekta Tajhiz, Iran), RevertAid First Strand cDNA Synthesis Kit (Thermo Scientific, USA).

### Methods

#### Synthesis and characterization of BG and TCP powders

The 45S5 bioactive glass (BG) powder with composition 45% SiO₂, 24.5% CaO, 24.5% Na₂O, and 5% P₂O₅ and β-tricalcium phosphate (β-TCP, Ca₃(PO₄)₂) were synthesized via sol-gel and aqueous precipitation methods respectively, as previously reported [42]. For the synthesis of 10 g of β-TCP, separate calcium and phosphate solutions were first prepared by dissolving 23.6 g of calcium nitrate tetrahydrate (Ca(NO₃)₂·4 H₂O, Merck, Germany) in 250 mL of deionized water (ddH₂O) and 8.8 g of diammonium hydrogen phosphate ((NH₄)₂HPO₄, Merck, Germany) in 250 mL of ddH₂O. The phosphate solution was then added dropwise to the calcium solution under continuous stirring while maintaining the pH at 6.5 using 0.1 M NaOH solution, monitored with a digital pH meter. The resulting suspension was centrifuged and lyophilized to obtain dry powder. For the synthesis of 10 g of 45S5 BG, 16.6 mL of tetraethyl orthosilicate (TEOS, C₈H₂₀O₄Si, Merck, Germany) was first added to 25 mL of deionized water under stirring along with 400 µL of 1 M HNO₃ solution as catalyst until a clear solution formed. Subsequently, 10.32 g of calcium nitrate (Merck, Germany), 6.72 g of sodium nitrate (Merck, Germany), and 1.44 mL of triethyl phosphate (TEP, C₆H₁₅O₄P, Sigma-Aldrich) were added sequentially to the initial solution with 45-minute intervals between additions under continuous stirring. The clear solution was then stored in a sealed container at room temperature for approximately one week until gelation occurred. The obtained gel was dried in an oven at 70 °C for 48 h followed by 120 °C for 24 h, and finally sintered at 700 °C for 3 h in an electric furnace to remove residual nitrates and obtain pure 45S5 BG powder. The particle size of the synthesized 45S5 BG powder, as characterized in our previous study [23], showed a nano-structured morphology with a wide distribution ranging from 171 nm to 1.25 μm and a peak diameter of 673 nm, as determined by dynamic light scattering (DLS) analysis.

To enhance the osteogenic properties of the scaffolds, zinc oxide (3, 9, 15% w/v) and cobalt oxide (1, 3, 5% w/v) were incorporated into the bioactive glass structure by adding zinc nitrate (Zn(NO₃)₂, Merck, Germany) and cobalt nitrate (Co(NO₃)₂, Merck, Germany) during synthesis, respectively. Table 1 presents the complete compositional details of the seven bioactive glass formulations developed in this study. The resulting doped powders underwent comprehensive characterization using the following engineering assessment methods:

X-ray diffraction (XRD) analysis was performed using a Siemens-Brucker D5000 diffractometer (40 kV/40 mA, Cu-Kα radiation) to examine the crystalline structure of synthesized bioactive glass (undoped, 1–5% Co-doped, and 3–15% Zn-doped) and β-TCP powders. Scans were conducted from 10° to 80° (2θ) with a step size of 0.02° and a dwell time of 1 s per step. Fourier-transform infrared (FTIR) spectroscopy (Nicolet NEXUS 670) was employed to analyze functional groups and chemical bonds in undoped, 1% CoO-doped, and 3% ZnO-doped bioactive glass and β-TCP samples. For FTIR measurements, powdered samples were mixed with potassium bromide (KBr) and pressed into pellets, with spectra collected at 4 cm⁻¹ resolution over 400–4000 cm⁻¹ (4 scans). Thermal analysis was conducted using an STA504 instrument (Bähr, Germany) to determine optimal sintering temperatures for undoped, 1% Co-doped, and 3% Zn-doped bioactive glass and β-TCP. Measurements were performed under argon atmosphere from 25 °C to 1300 °C at heating rates of 10–70 °C/min.

**Table 1:** Bioactive glasses composition synthesized in this study

Sample code	SiO₂	CaO	Na₂O	*P*₂O₅	ZnO	CoO
45S5 BG	45	24.5	24.5	5	0	0
3%Zn-45S5 BG	45	24.5	21.5	5	3	0
9%Zn-45S5 BG	45	24.5	15.5	5	9	0
15%Zn-45S5 BG	45	24.5	9.5	5	15	0
1%Co-45S5 BG	45	24.5	23.5	5	0	1
3%Co-45S5 BG	45	24.5	21.5	5	0	3
5%Co-45S5 BG	45	24.5	19.5	5	0	5

#### Fabrication of the scaffolds via robocasting technique

Three scaffold variants were fabricated: (1) undoped bioactive glass/tricalcium phosphate (BG/TCP), (2) zinc-doped BG/TCP (3–15% Zn), and (3) cobalt-doped BG/TCP (1–5% Co). As detailed previously, ion doping was performed during BG powder synthesis, and all composites maintained a 50:50 w/w BG: TCP ratio based on prior optimization [39]. The printing ink was formulated by sequentially adding gelatin (0.5% w/v), PVA (10% w/v), Tween 60 surfactant (0.25% w/v), and equal portions of BG/TCP powders (19% w/v each) to deionized water, followed by 1-hour magnetic stirring. This yielded an ink with optimal rheological properties as established in our earlier work [23]. The homogenized ink was loaded into syringes for robocasting (ABTIN I, Abtin Teb Fanavar, Iran) controlled by Repetier-Host software. Scaffolds were printed using a nozzle diameter of 510 μm, a layer height of 500 μm, and a printing speed of 4 mm/s. This process produced 15 × 15 × 5 mm³ scaffolds through layer-by-layer deposition. Final sintering at 1150 °C (1 h dwell time) followed the established thermal profile to achieve target mechanical strength [23].

### Primary analyses to find the optimum doping value for Zn and Co in BG/TCP scaffolds

#### Evaluation of scaffold surface morphology by SEM observation

The microstructure of 3D-printed scaffolds was analyzed using scanning electron microscopy (SEM) (Philips XL30 SEM) to evaluate morphological features, including pore architecture and surface topography. Prior to imaging, scaffold surfaces were sputter-coated with a 15 nm gold layer to ensure conductivity. Observations were conducted at an accelerating voltage of 15 kV across multiple magnifications (50× to 5000×), enabling comprehensive assessment of both macroporous networks (100–500 μm) and micro surface features (< 10 μm). Representative images were systematically captured to document: (1) interconnective pore geometry, (2) strut morphology, and (3) surface roughness at the material-tissue interface.

#### Evaluation of cytotoxicity by MTT assay

The indirect cytotoxicity of scaffolds was assessed using an MTT assay according to ISO 10993-5 guidelines. All scaffold groups (undoped, 1–5% Co-doped, and 3–15% Zn-doped BG/TCP) underwent sequential sterilization via UV irradiation (30 min per side) followed by dry heat treatment (250 °C for 2 h). Sterilized scaffolds were individually immersed in 1 mL of high-glucose DMEM in 24-well plates and conditioned for 1, 3, and 5 days in a humidified incubator (37 °C, 5% CO₂) to obtain extract media. Human adipose-derived mesenchymal stem cells (hADMSCs) were isolated from lipoaspirate samples through enzymatic digestion with 0.1% collagenase type (I) (37 °C, 45 min) and expanded through passage 3–5 in α-MEM supplemented with 10% fetal bovine serum. For cytotoxicity testing, 1 × 10⁴ hADMSCs per well were seeded in 96-well plates and allowed to adhere for 24 h. At each time point, the culture medium was replaced with 100 µL of scaffold-conditioned medium (*n* = 6 replicates per group). Following 24 h of exposure, cells were incubated with 100 µL of MTT solution (0.5 mg/mL in PBS) for 4 h at 37 °C. The formazan crystals formed were subsequently solubilized with 100 µl of DMSO per well with 10 min of orbital shaking. Absorbance was measured at 570 nm with a 630 nm reference using a microplate reader (BioTek Synergy HT), with results normalized to negative controls containing culture medium alone. Viability percentages were calculated relative to positive controls (cells in standard growth medium).

#### Evaluation of ion release by ICP-OES

To characterize the release kinetics of bioactive ions (Ca, P, Si, Zn, and Co) from the scaffolds, samples from all experimental groups (*n* = 4 independent scaffolds per group per time point) were first rinsed with phosphate-buffered saline (PBS) to remove surface residues. Scaffolds were then immersed in Dulbecco’s Modified Eagle Medium (DMEM) at a concentration of 0.1 mg/mL and incubated under standard cell culture conditions (37 °C, 5% CO₂) for 3, 7, 14, and 21 days. At each predetermined time point, the culture media were carefully collected and immediately stored at -20 °C to preserve ion concentrations until analysis. Quantitative measurement of released ions was performed using inductively coupled plasma optical emission spectrometry (ICP-OES, Model 7300DV, PerkinElmer), with calibration curves established using certified reference materials for each element. The instrument’s detection limits were 0.01 ppm for Ca and P, 0.005 ppm for Si, and 0.001 ppm for both Zn and Co, ensuring accurate quantification across the expected concentration ranges.

### Engineering characterization of the selected scaffolds

#### XRD analysis

To assess composition and crystalline structure, the scaffolds were ground into powder and analyzed using X-ray diffraction (XRD) (Siemens-Brucker D5000 diffractometer, 40 kV/40 mA, Cu-Kα radiation, λ = 1.54 Å). The scanning range was set from 10° to 80° with a step size of 0.02° and a dwell time of 1 s per step.

#### Mechanical property assessment

The compressive strength of the scaffolds was evaluated according to the ASTM F2150 standard guide for biomaterial scaffolds. The test was performed on selected scaffolds (*n* = 5 per group) using a universal testing machine (SANTAM, Iran) equipped with a 50 kN load cell at a crosshead speed of 0.5 mm/min. Samples were compressed to 40% of their original height, with compressive strength determined from the maximum load observed in the stress-strain curve. The elastic modulus was calculated as the slope of the linear elastic region in the stress-strain curve.

#### Bioactivity assessment in simulated body fluid (SBF)

To evaluate bioactivity, selected scaffolds were immersed in 5 mL of simulated body fluid (SBF). The SBF was prepared according to the Kokubo recipe [49] with ion concentrations (in mM: Na⁺ 142.0, K⁺ 5.0, Mg²⁺ 1.5, Ca²⁺ 2.5, Cl⁻ 147.8, HCO₃⁻ 4.2, HPO₄²⁻ 1.0, SO₄²⁻ 0.5) nearly equal to those of human blood plasma, and buffered to a pH of 7.40 at 37 °C. Scaffolds were incubated for 3, 7, and 14 days under constant agitation in a shaker incubator at 37 °C. After each time period, samples were removed from SBF, rinsed with deionized water, and dried completely at room temperature. Surface mineralization was characterized using scanning electron microscopy (SEM) to assess calcium phosphate layer formation, followed by X-ray diffraction (XRD) analysis to identify crystalline phases.

### Biological characterization of selected scaffolds

#### Isolation of human adipose-derived mesenchymal stem cells (hADMSCs) and characterization

Human adipose-derived mesenchymal stem cells (hADMSCs) were isolated from abdominal adipose tissue (two 7 × 7 cm pieces) obtained with informed consent from a 32-year-old overweight woman undergoing abdominoplasty surgery, with samples transported in Falcon tubes containing Hanks’ culture medium. For isolation, the adipose tissue was minced and transferred to a 50 mL Falcon tube containing an equal volume of Hanks’ medium supplemented with 3× penicillin/streptomycin and 2% amphotericin B, vortexed for 2–3 min, then centrifuged at 2,500 rpm for 15 min. The resulting pellet was digested with an equal volume of 3 mg/mL collagenase type (I) (prepared in Hanks’ medium and filtered) and incubated at 37 °C with 5% CO₂ for 2–3 h. After digestion, the suspension was centrifuged at 2,500 rpm for 15 min, the supernatant removed, and the cell pellet resuspended in 5 mL of DMEM/F12 supplemented with 10% FBS, 2% penicillin/streptomycin, and 2% amphotericin B. The cell suspension was sequentially filtered through 70 μm and 40 μm strainers before being seeded in culture flasks and expanded to confluence for subsequent differentiation assays.

To confirm the mesenchymal phenotype, flow cytometry was performed using 1 × 10⁵ trypsinized cells resuspended in PBS. Cells were stained with 5–10 µL of fluorescently conjugated antibodies against mesenchymal markers (CD90, CD73, CD105) and hematopoietic lineage markers (CD45, CD34), followed by 30-minute incubation at 4 °C in the dark. After washing with PBS, cells were fixed in 4% paraformaldehyde and analyzed using a FACSCalibur flow cytometer (Becton Dickinson). The mesenchymal identity was confirmed by positive expression of CD90/CD73/CD105 (> 95% positive) and negative expression of CD45/CD34 (< 2% positive). The flow cytometry device comprises three main components: (1) a fluidics system that transports cells in suspension, (2) a laser system where individual cells pass through the light beam for interrogation, and (3) an electronic detection system that analyzes scattered and fluorescent light signals. Cells were gated based on size and density parameters in the resulting plots, with the isotope control (negative control) region identified first. Raw data were processed using FLOWJo software to determine marker expression levels through the flow cytometry analysis system.

#### Evaluation of cell attachment on the scaffolds

The selected scaffolds were sterilized at 250 °C and placed in a 24-well plate. Each well was pre-wetted with 1 mL DMEM medium and incubated for 2 h at 37 °C. Meanwhile, hADMSCs were trypsinized, isolated, and counted. Subsequently, 30 × 10³ cells were seeded onto each scaffold surface, followed by careful addition of 1 mL fresh DMEM medium along the well wall. After 3 days of incubation, cell-scaffold constructs were fixed with 4% paraformaldehyde (40 min), then dehydrated through an ethanol gradient (30%, 50%, 70%, 90%, and twice 100%, 5 min each). Samples were air-dried overnight, sputter-coated with gold, and examined by SEM to evaluate cell attachment morphology.

#### Gene expression analysis by qRT-PCR

Following the 21-day differentiation period, cell viability was assessed using trypan blue staining, and an equal cell number (1 × 10⁵) was used for osteogenic marker analysis. Total RNA was extracted using a commercial RNA isolation kit (Yekta Tajhiz, Iran) following the manufacturer’s protocol. RNA purity and concentration were quantified using a NanoDrop ND-1000 spectrophotometer (Thermo Scientific, USA), with acceptable purity defined by A₂₈₀/A₂₆₀ ratios of 1.8-2.0 and A₂₆₀/A₂₃₀ ratios > 2.0, confirming the absence of protein contamination (A₂₈₀) or organic solvents/phenol (A₂₃₀). Subsequent cDNA synthesis and qRT-PCR were performed to evaluate expression levels of osteocalcin (OCN) and collagen type I (Col-1) genes.

Following RNA extraction, cDNA was synthesized using the RevertAid First Strand cDNA Synthesis Kit (Thermo Scientific, USA) according to the manufacturer’s protocol. Gene-specific primers were designed using Oligo-7 software and commercially synthesized (Pishgam, Iran; sequences provided in Table 2). qPCR reactions were performed in a final volume of 20 µL containing: 1 µL cDNA template, 0.8 µL each of forward and reverse primers (10 µM), 10 µL SYBR Green PCR Master Mix, and 7.4 µL nuclease-free water. For each experimental group, RNA was extracted from three independent cell culture experiments (*n* = 3 biological replicates). Each cDNA sample was then analyzed in triplicate during the qPCR run (three technical repetitions). The glyceraldehyde-3-phosphate dehydrogenase (GAPDH) gene served as the endogenous control for normalization, with relative gene expression calculated using the 2^(-ΔΔCt) method.

**Table 2:** Primer sequences used for real time PCR in the current study

Gene name	Oligo sequence (5’→3’)	Base no
Human GAPDH-F	CCT CAA GAT CAT CAG	22
Human GAPDH-R	CCA TCA CGC CAC AGT	20
Human COL1A1-F	GCC AAG ACG AAG ACA	21
Human COL1A1-R	TCA CGT CAT CGC ACA	21
Human OCN-F	GGT GCA GCC TTT GTG	19
Human OCN-R	TCA GCC AAC TCG TCA	21

#### Immunocytochemical analysis of osteogenic markers

Following the 21-day differentiation period, cells were washed twice with PBS and fixed with 4% paraformaldehyde (pH 7.4) for 10 min at room temperature. After PBS washing, cell membranes were permeabilized with 0.25% Triton X-100 in PBS (10 min). Non-specific binding was blocked using 1% BSA with 22.54 mg/mL glycine in PBST (0.1% Tween-20) for 30 min. Cells were then incubated overnight at 4 °C with primary antibodies against collagen type I (COL1A1, sc-59772) and osteopontin (OPN, #66377) in blocking solution. After PBST washes, samples were incubated with Alexa Fluor-conjugated secondary antibodies (1:500 dilution) for 2 h at room temperature (dark conditions). Nuclei were counterstained with DAPI (0.5 µg/mL, 1 min). For each experimental group, fluorescent images were acquired from five random fields per well using an Olympus IX71 inverted microscope. The experiment was performed with three independent biological replicates (*n* = 3).

#### Alkaline phosphatase (ALP) activity assessment

During osteogenic differentiation (7- and 14-day timepoints), cell culture supernatants were collected every 2–3 days from three independent differentiation experiments (*n* = 3 biological replicates), aliquoted in foil-wrapped microtubes, and stored at -80 °C until analysis. For ALP quantification, 50 µL of supernatant was mixed with 200 µL of 10 mM p-nitrophenyl phosphate (pNPP) substrate solution and incubated for 30 min at 37 °C. The enzymatic reaction was stopped with 50 µL of 2 N NaOH, and absorbance was measured at 405 nm using a microplate reader. Each sample was measured in triplicate (*n* = 3 technical replicates). ALP activity (U/mL) was calculated against a p-nitrophenol standard curve, normalized to total protein content when applicable.

#### Mineralization assessment by Alizarin red staining

Following 21 days of osteogenic differentiation, cells were washed with PBS and fixed with 10% neutral buffered formalin (1 h). After fixation, samples were stained with 2% Alizarin Red S solution (pH 4.2 ± 0.1) for 5 min at room temperature to visualize calcium deposits. Excess dye was removed by sequential washes with: (1) distilled water to remove unbound dye, (2) 100% acetone for 30 s, and (3) acetone: xylene (1:1) for complete dehydration. Mineralized nodules appeared as distinct red-orange deposits. For quantification, images were captured from five random fields per well using a light microscope. The area of mineralization was quantified based on staining intensity using ImageJ software (National Institutes of Health, USA). This analysis was performed on samples from three independent differentiation experiments (*n* = 3 biological replicates).

### Statistical analysis

Data are presented as mean ± standard deviation (SD). The number of independent replicates (n) for each experiment is stated in the respective method sections above. Statistical analysis was performed using GraphPad Prism software. For comparisons between multiple groups, one-way analysis of variance (ANOVA) was used, followed by Tukey’s post hoc test for pairwise comparisons. A p-value of less than 0.05 (*p* < 0.05) was considered statistically significant. Flow cytometry data analysis was performed using FlowJo software. Quantification of immunocytochemistry results was performed using ImageJ software.

## Results

### Characterization of synthesized BG and TCP powders

**Fig. 1:**
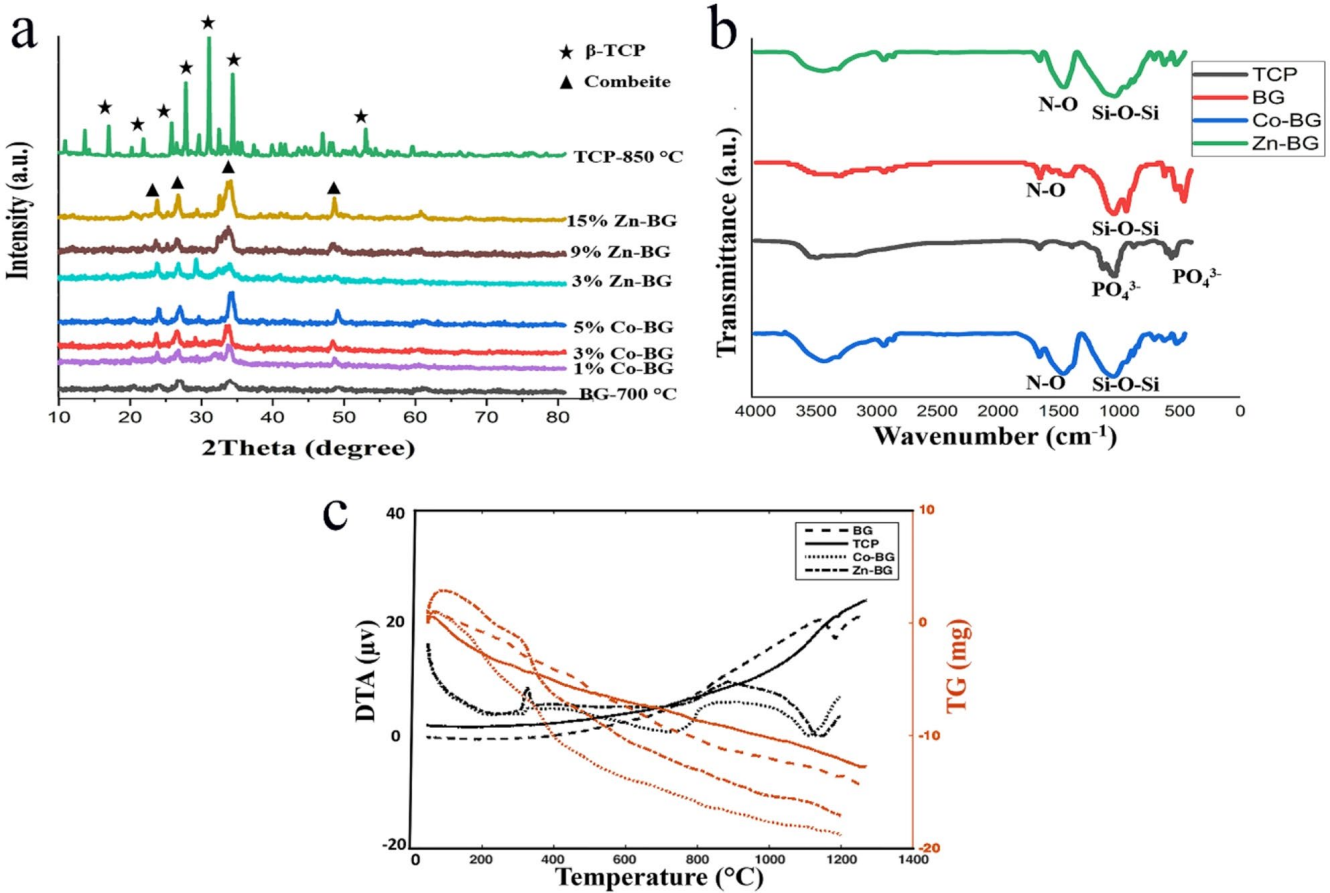
Results acquired from characterization of synthesized powders: **a**- XRD analysis of powders (BG, TCP, 3,9,15% Zn-BG and 1,3,5% Co-BG), **b**- FTIR analysis of powders (BG, TCP, 3% Zn-BG and 1% Co-BG), **c**- DTA/TG analysis of powders (BG, TCP, 3% Zn-BG and 1% Co-BG)

Figure 1 presents the XRD, FTIR, and STA (DTA/TG) analyses of synthesized undoped, Zn-doped (3, 9, 15 wt%), and Co-doped (1, 3, 5 wt%) BG powders along with TCP. The XRD diffractograms (Fig. 1a) reveal that while bioactive glasses typically exhibit amorphous structures, high-temperature sintering induced partial crystallization in the prepared BG, resulting in a semi-crystalline structure. Phase analysis identified characteristic diffraction peaks between 20–30° and 30–40° 2θ angles, which matched the reference pattern for combeite (Na₂Ca₂Si₃O₉; ICDD PDF #00-023-0671). This crystalline phase formation was confirmed through comparison with standard calcium silicate compounds in the ICDD database.

The XRD analysis of precipitated TCP powder calcined at 850 °C confirmed exclusive formation of the β-TCP crystalline phase, with all diffraction peaks matching the standard β-tricalcium phosphate reference pattern (JCPDS #09-0169). The sharp, well-defined peaks indicate high crystallinity achieved through the precipitation synthesis route.

The FTIR spectra of synthesized powders (Fig. 1b) revealed characteristic absorption bands confirming their chemical compositions. For β-TCP, distinct phosphate vibrational modes appeared at 500–605 cm⁻¹ (ν₄ bending vibrations of PO₄³⁻) and 900–1200 cm⁻¹ (ν₁/ν₃ stretching vibrations of PO₄³⁻), consistent with crystalline β-tricalcium phosphate (JCPDS #09-0169). Additional bands at 1650 cm⁻¹ (H-O-H bending) and 3640 cm⁻¹ (O-H stretching) indicated surface-adsorbed moisture, a common feature in bioceramic powders.

Figure 1-c presents the simultaneous DTA/TG analysis of undoped BG, Zn-BG (3–15%), Co-BG (1–5%), and β-TCP. The TG curves revealed three-stage weight losses: (1) 30–200 °C (physiosorbed water), (2) 200–600 °C (organic residue combustion), and (3) 600–800 °C (nitrate decomposition). Undoped BG exhibited a characteristic endothermic peak at 1140–1220 °C in the DTA curve, corresponding to its glass transition temperature (Tg) - a critical parameter for sintering temperature selection. Notably, doping reduced Tg significantly: Co-BG showed a 50 °C depression (1090–1170 °C) while Zn-BG demonstrated a more pronounced 70 °C decrease (1070–1150 °C). This Tg reduction enables lower sintering temperatures, effectively suppressing excessive crystallization during thermal processing. The observed melting point depression follows the typical behavior of network modifiers in silicate glasses, where Zn²⁺ and Co²⁺ ions disrupt the Si-O-Si connectivity more efficiently than Na⁺/Ca²⁺ in 45S5 BG.

The STA analysis of TCP powder (Fig. 1-c) revealed characteristic thermal transitions through complementary TG and DTA curves. The TG profile showed a continuous mass loss (~ 5–7%) up to 1000 °C, attributed to the elimination of residual hydroxyl groups and carbonate contaminants from the precipitation synthesis. The DTA curve exhibited a sharp endothermic peak at 1190 °C, corresponding to the β→α phase transformation of TCP - a critical thermal event that dictates the maximum sintering temperature to maintain phase purity. This allotropic transition temperature aligns with standard β-TCP references (JCPDS #09-0169) and serves as a key parameter for optimizing thermal processing conditions.

### Optimization of Zn and Co doping concentrations in BG/TCP scaffolds via preliminary analyses

#### Surface morphology of the scaffolds by SEM

**Fig. 2:**
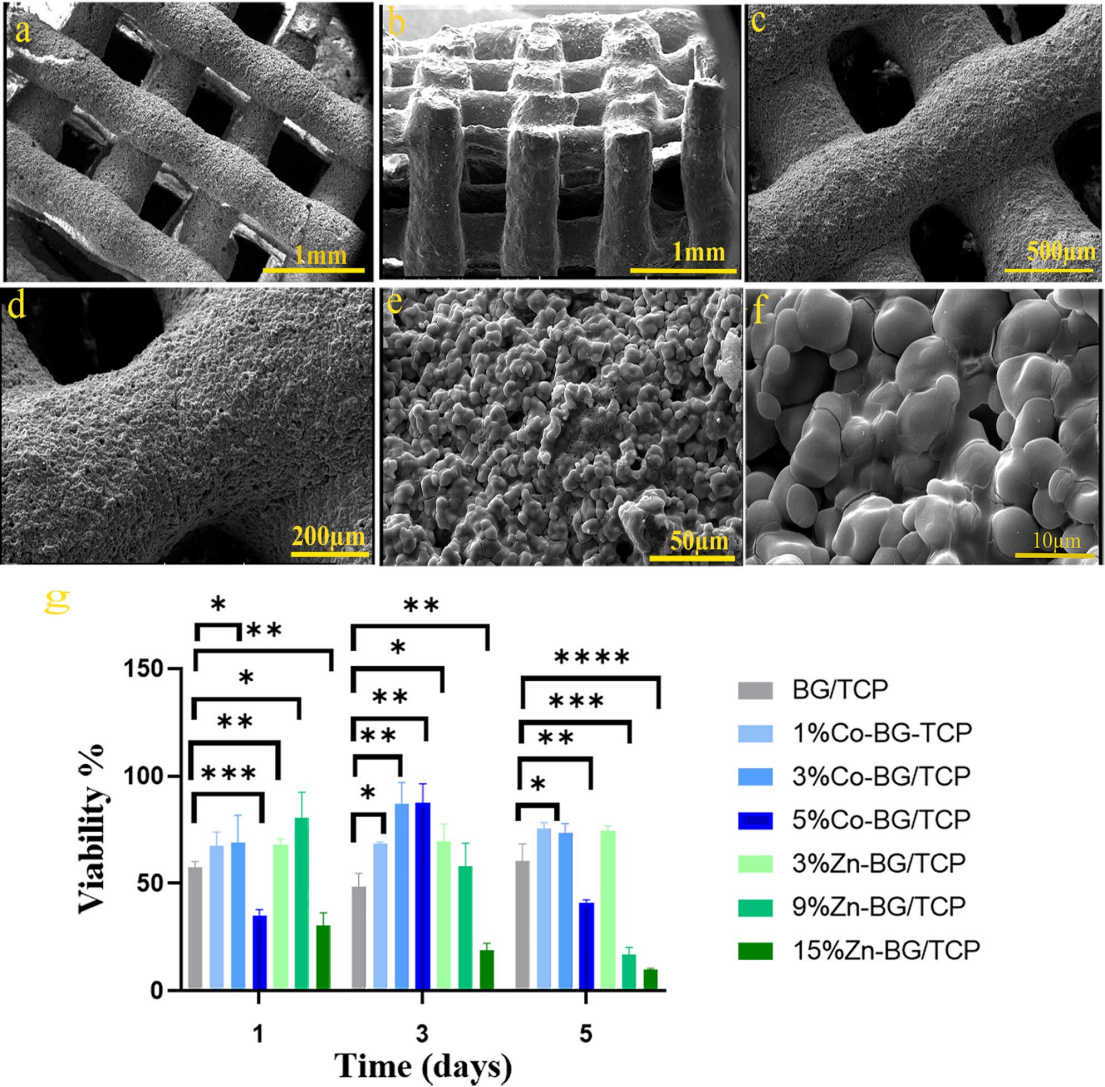
(**a**-**f**) Morphology of BG/TCP scaffold at different magnifications prepared in this study observed by SEM, (**g**) MTT assay results of prepared BG/TCP scaffolds with different doping amounts of Zn and Co at different times. (p- value*<0.1, p-value**< 0.01, p-value***<0.001, p-value****<0.0001)

SEM analysis (Fig. 2a-f) revealed the optimized porous architecture of BG/TCP scaffolds with uniform, interconnected channels measuring 475–589 μm in diameter, achieving a total porosity of 40% as previously reported [40]. This hierarchical pore structure falls within the ideal range for bone tissue regeneration, facilitating vascularization and cell infiltration. At higher magnifications (Fig. 2f), the sintered microstructure demonstrates effective particle coalescence through liquid phase formation, a critical factor enhancing mechanical integrity. The observed melting and bonding of BG particles confirm optimal sintering conditions were achieved, as evidenced by the continuous glassy matrix surrounding TCP grains.

#### Evaluation of cytotoxicity by MTT assay

To evaluate the effects of scaffold composition and ion doping concentrations on mesenchymal stem cell viability, indirect MTT assays were conducted over 1, 3, and 5 days (Fig. 2g). Notably, the 3% Zn-BG/TCP and both 1% and 3% Co-BG/TCP groups demonstrated significantly higher cell viability compared to undoped controls at all timepoints (*p* < 0.05). These results indicate that controlled incorporation of Zn²⁺ and Co²⁺ ions into the BG structure enhances cellular survival, suggesting beneficial biological effects of the doped compositions.

Cell viability in the undoped BG/TCP group showed a transient reduction on day 3 compared to day 1 (*p* > 0.05), likely attributable to initial ion burst release, localized pH elevation, and metabolic byproduct accumulation. By day 5, viability rebounded, potentially due to: (1) possible hydroxyapatite layer formation improving surface biocompatibility, (2) stabilization of ion release kinetics, and (3) cellular adaptation to the microenvironment. Notably, the 3% Zn-BG/TCP and 1–3% Co-BG/TCP groups demonstrated significantly enhanced viability versus controls (*p* < 0.05), whereas higher dopant concentrations (9–15% Zn, 5% Co) exhibited cytotoxic effects, as evidenced by 20–25% reduced hADMSC survival (Fig. 2g). This concentration-dependent response guided selection of 3% Zn-BG/TCP and 1% and 3% Co-BG/TCP scaffolds for subsequent studies, optimizing the balance between bioactive stimulation and cellular tolerance.

#### ICP-OES analysis of ionic release profiles

**Fig. 3:**
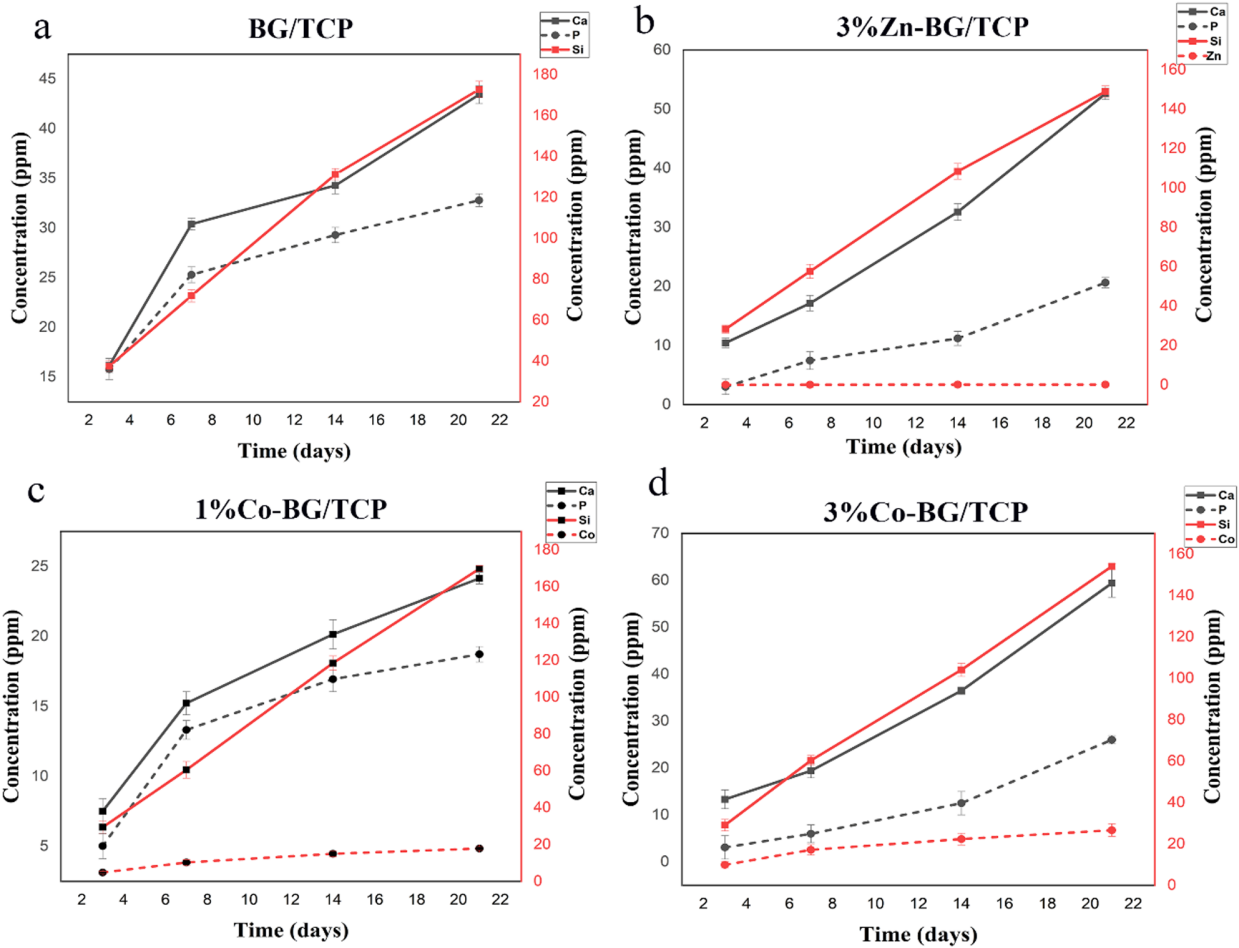
Evaluation of ion release by ICP-OES assay: **a**- release of ions from BG/TCP scaffold, **b**- release of ions from 3%Zn-BG/TCP scaffold, **c**- release of ions from 1% Co-BG/TCP, **d**- release of ions from 3% Co-BG/TCP

The 21-day release kinetics of Si, Ca, P, Zn, and Co ions from composite scaffolds (BG/TCP, 3% Zn-BG/TCP, 1% Co-BG/TCP, and 3% Co-BG/TCP) in DMEM were quantified using ICP-OES (Fig. 3). All groups exhibited progressive ion accumulation over the study period, with particularly pronounced silicate (Si⁴⁺) release demonstrating an initial rapid elution phase followed by sustained release - consistent with established literature demonstrating silicon’s significant stimulatory effects on mesenchymal cell proliferation and differentiation [15]. While both Zn²⁺ and Co²⁺ (recognized osteogenic factors) showed concentration-dependent release patterns, Co²⁺ elution rates substantially exceeded Zn²⁺, reaching final concentrations of 18 ppm (1% Co-BG/TCP) and 26 ppm (3% Co-BG/TCP). Notably, the 3% Co concentration surpassed the 20 ppm viability threshold associated with cytotoxic effects [38], corroborating our MTT findings. Consequently, the 1% Co-BG/TCP (maintaining subtoxic Co²⁺ levels) and 3% Zn-BG/TCP (exhibiting optimal Zn²⁺ release kinetics) were selected for subsequent characterization as they optimally balanced bioactive ion delivery with cellular compatibility.

### Engineering characterization of selected scaffolds

#### XRD analysis

**Fig. 4:**
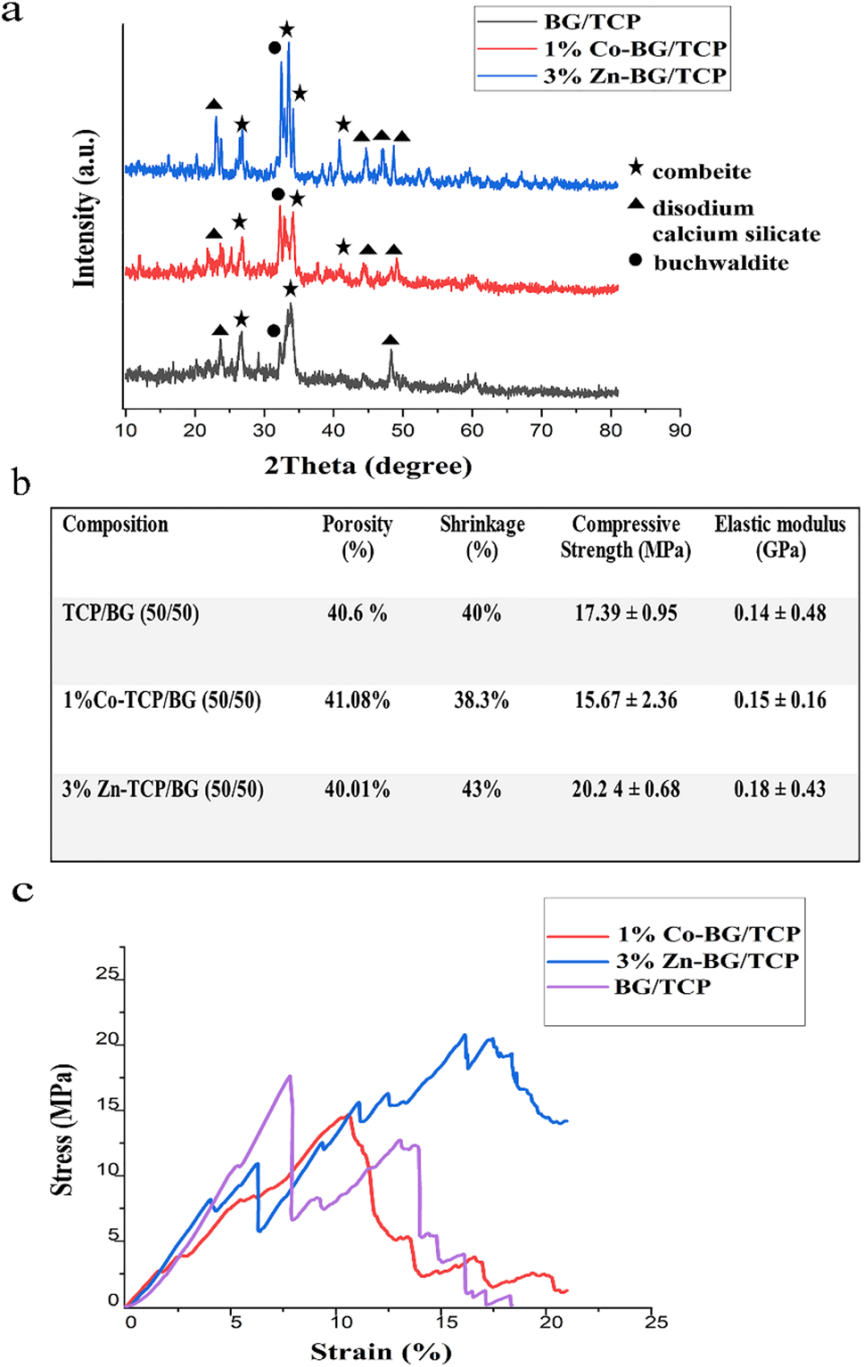
**a**-XRD diffractogram acquired from the selected scaffolds (BG/TCP, 3%Zn-BG/TCP, and 1%Co-BG/TCP), **b**- Mechanical indices obtained in mechanical evaluation of BG/TCP, 3%Zn-BG/TCP, and 1%Co-BG/TCP scaffold under compression, **c**- Sample stress-strain curves obtained from the prepared scaffolds under compressive test (BG/TCP, 3%Zn-BG/TCP, and 1%Co-BG/TCP scaffold). The characteristic jagged appearance of the curves is not electronic noise but reflects the intrinsic brittle fracture behavior of the ceramic scaffolds, involving sequential micro-cracking and strut failure during compression

Figure 4a presents the XRD patterns of 1% Co-BG/TCP and 3% Zn-BG/TCP scaffolds, revealing characteristic peaks corresponding to both silicate and calcium phosphate phases. The diffraction patterns confirm the presence of combeite (Na₄Ca₄Si₆O₁₈), disodium calcium silicate (Na₂CaSiO₄), and buchwaldite (NaCaPO₄), demonstrating the formation of a composite structure through reactive sintering. Notably, the doped scaffolds exhibited sharper diffraction peaks compared to undoped controls, indicating enhanced crystallinity while maintaining the same phase composition. This preservation of phase identity despite increased crystallinity suggests that Zn²⁺ and Co²⁺ incorporation primarily affects crystal growth rather than phase formation, with dopant ions likely occupying network-modifying positions in the glass-ceramic structure.

#### Mechanical characteristics

Figure 4b-c presents the comprehensive characterization of the selected scaffolds, including composition, porosity, shrinkage, mechanical strength, and stress-strain behavior. All scaffolds maintained a consistent total porosity of ~ 40%according to previous study [39], with post-sintering shrinkage ranging from 38.3 to 43%. This shrinkage demonstrated a direct correlation with mechanical properties, as increased densification led to enhanced compressive strength (15.67–20.24 MPa) - values well within the range of native cancellous bone [46].

#### Bioactivity evaluation of the scaffolds

**Fig. 5:**
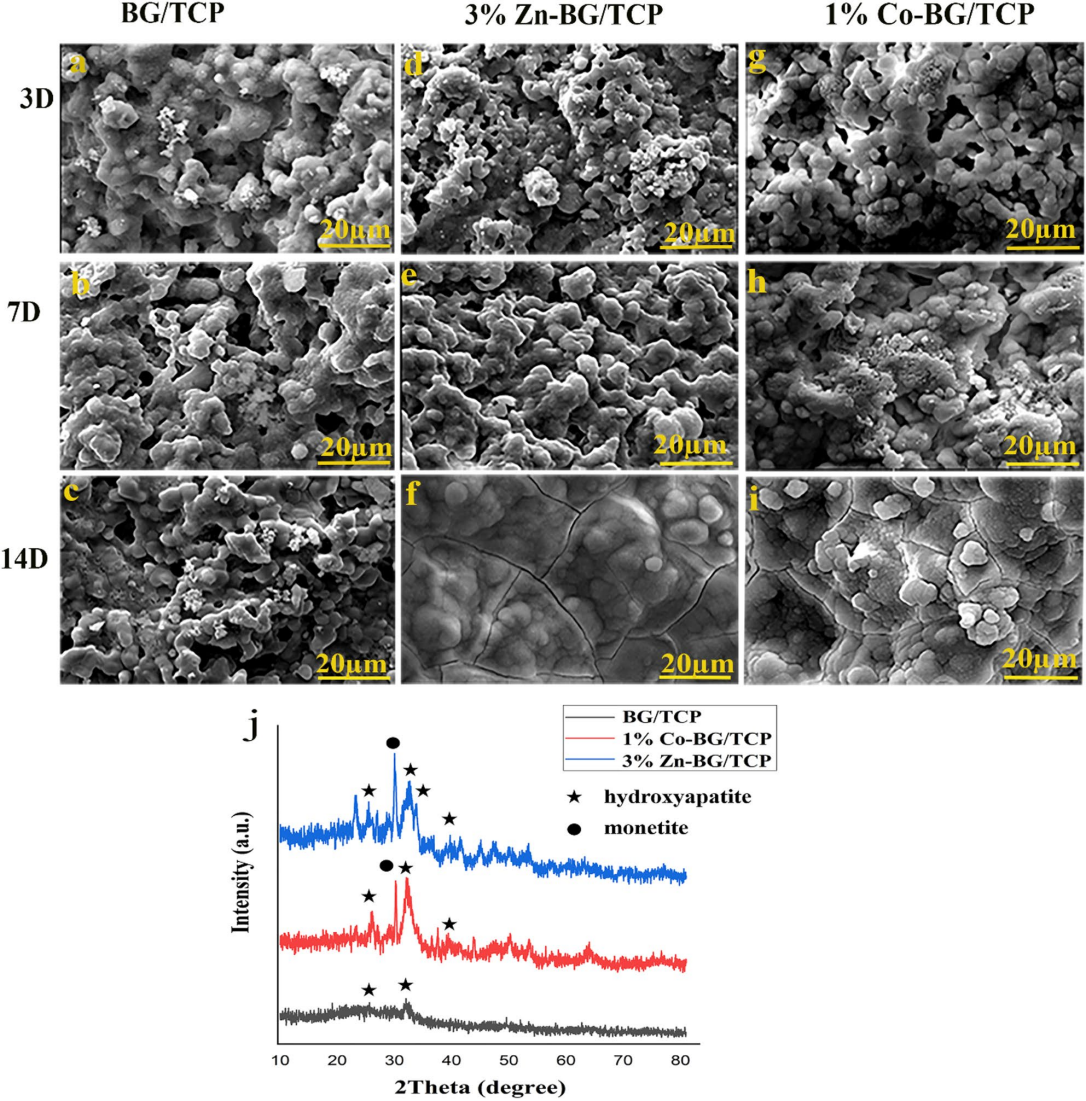
Bioactivity analysis of the selected scaffolds after 3,7,14 days soaking in SBF, **a**-**c**: surface morphology of BG/TCP scaffold, **d**-**f**: surface morphology of 3%Zn-BG/TCP scaffold, surface morphology of 1%Co-BG/TCP scaffold, **j**- XRD diffractogram acquired from the surface of the soaked scaffolds in SBF after 14 days showing formation of new minerals layer on the surface

The results related to bioactivity were shown in Fig. 5. The bioactivity of the selected scaffolds was evaluated in vitro by immersing them in SBF solution for periods of 3, 7, and 14 days. The surface morphology of composite scaffolds after immersion in SBF solution at different times was shown in Fig. 5 (a-i). As can be seen, after 3 days of soaking, small and scattered particles were deposited on the surfaces of the composite scaffolds. On the 7th day, more mineralization cores are observed, which are distributed in almost the same way, and on the 14th day, the surface of the selected scaffolds is completely covered, although the mineral layer covering the surface of Zn-BG/TCP and Co-BG/TCP scaffolds is thicker compared to BG/TCP.

XRD characterization of scaffolds following 14-day SBF immersion (Fig. 5) confirmed hydroxyapatite (HA) formation through identification of characteristic diffraction peaks (JCPDS #09-0432) on all scaffold types. The doped compositions (Zn-BG/TCP and Co-BG/TCP) exhibited additional monetite (CaHPO₄; JCPDS #09–0080) phases, suggesting alternative mineralization pathways mediated by dopant ions.

### Biological characterizations

#### Flow cytometry

**Fig. 6:**
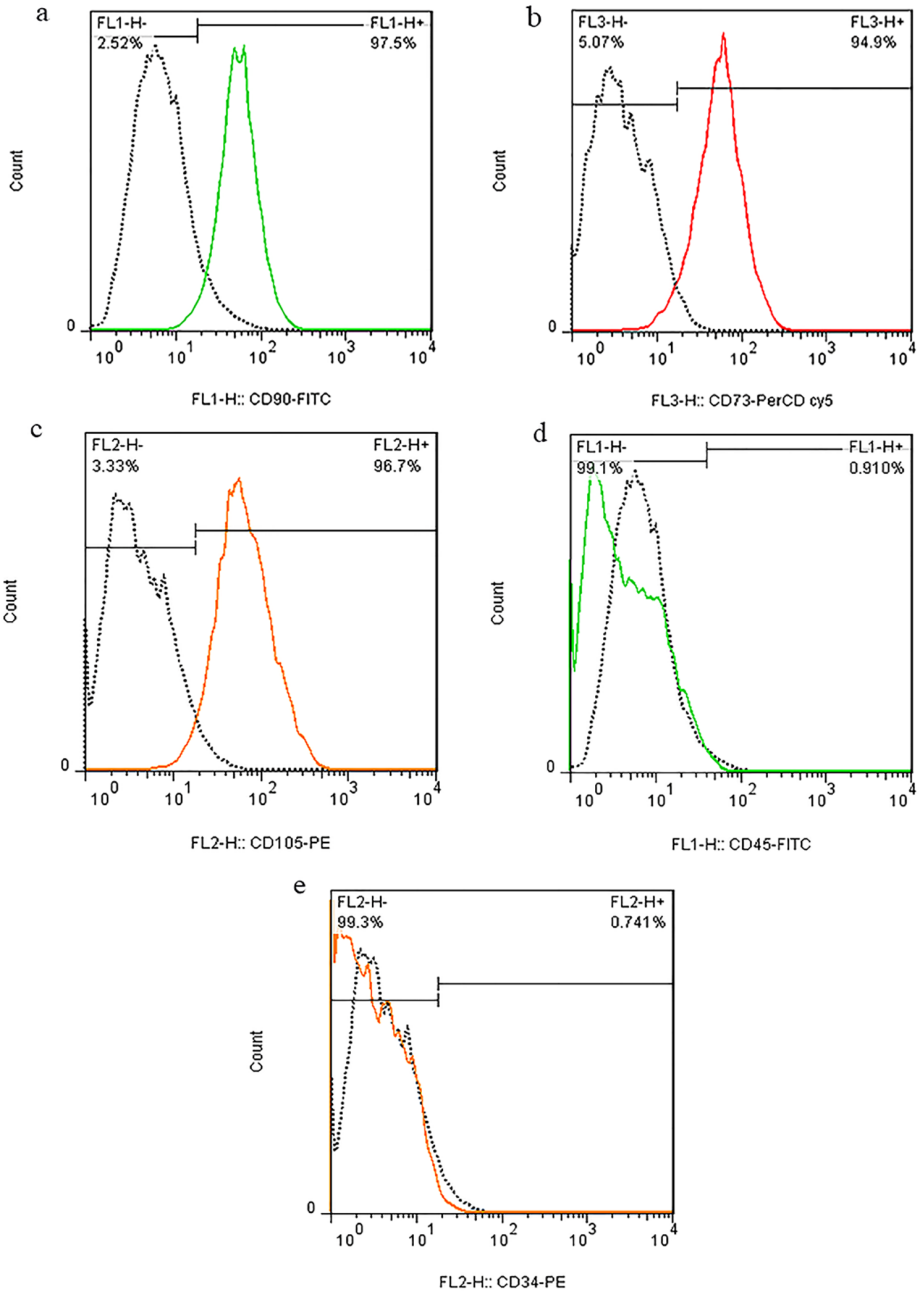
The expression of mesenchymal markers (CD 90, CD73, and CD105) and non-mesenchymal markers (CD 45 and CD 34) in the extracted hADMSCs (human adipose-derived mesenchymal stem cells) using flowcytometry analysis

To verify the mesenchymal stem cell (MSC) identity of human adipose-derived cells, flow cytometry was performed using antibodies against standard MSC markers (CD90, CD73, CD105) and hematopoietic lineage markers (CD34, CD45). Cells were analyzed and data were processed with FlowJo software (vX.X; Tree Star). Histograms revealed high expression of MSC-associated markers (CD90: 97.5%; CD73: 94.9%; CD105: 96.7%) and negligible expression of hematopoietic markers (CD34, CD45; <2% positivity), consistent with ISCT criteria for MSCs. In Fig. 6, the X-axis depicts logarithmic fluorescence intensity of each marker, while the Y-axis represents cell counts, demonstrating a homogeneous population devoid of contaminating hematopoietic cells.

#### Cell morphology and adhesion on the selected scaffolds

**Fig. 7:**
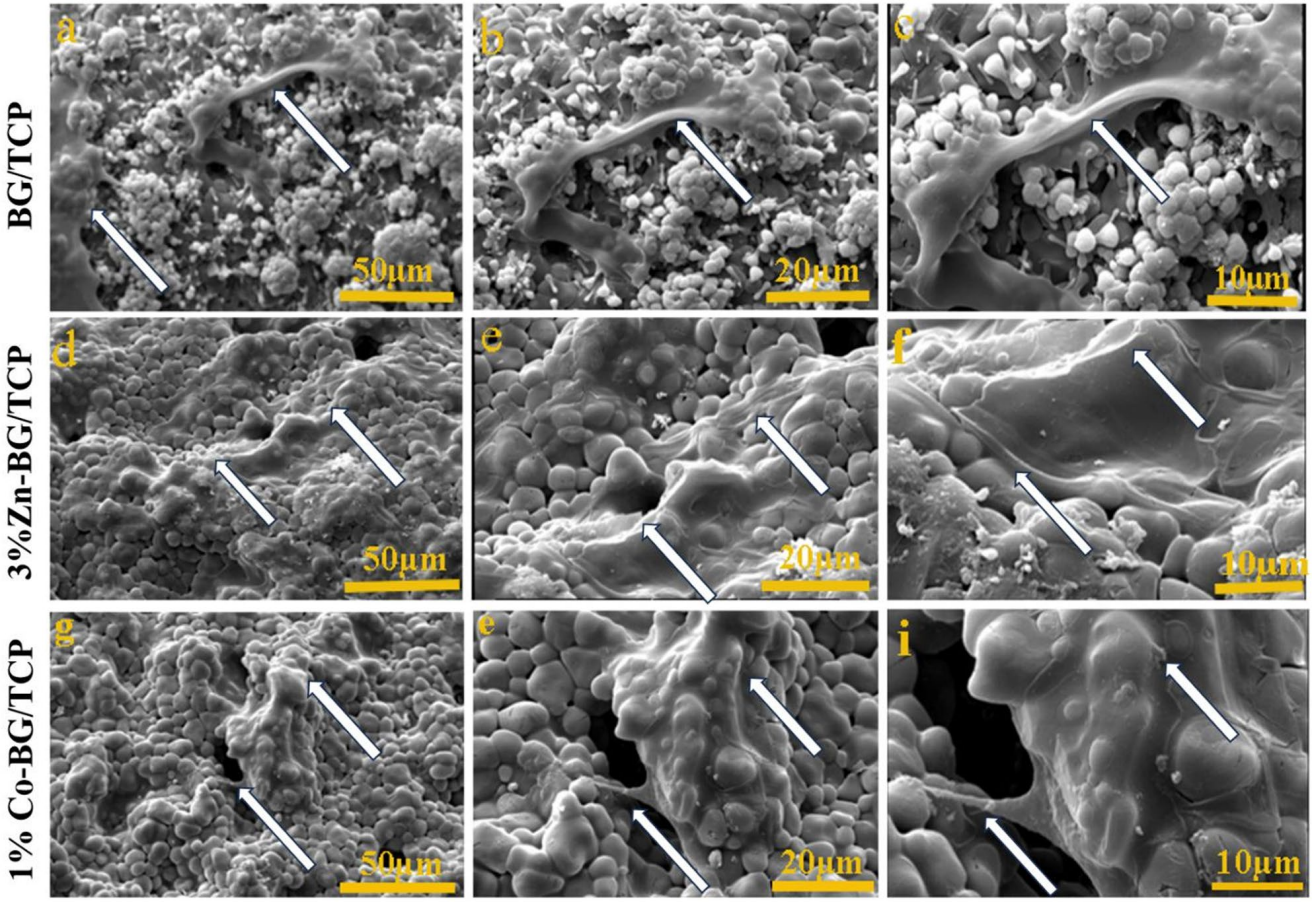
Morphology and attachment of hADMSCs seeded on selected composite scaffolds at different magnifications; (**a**-**c**) attachment of hADMSCs on the surface of the BG/TCP scaffold, (**d**-**f**) 3%Zn-BG/TCP scaffold, (**g**-**i**) 1%Co-BG/TCP scaffold taken by SEM microscope

SEM analysis at varying magnifications (Fig. 7) revealed the morphology and adhesion behavior of human adipose-derived mesenchymal stem cells (hADMSCs) cultured on three scaffold types: BG/TCP, Zn-BG/TCP, and Co-BG/TCP. The SEM images demonstrated robust cell attachment, with cytoplasmic extensions anchoring firmly to both the surface and pore walls of all scaffolds, indicating favorable chemical and topographical properties for cell adhesion. Notably, hADMSCs exhibited more extensive surface coverage on Zn-BG/TCP and Co-BG/TCP scaffolds compared to BG/TCP, suggesting enhanced biocompatibility or adhesion potential in the doped scaffolds.

#### Assessment of gene expression by real time PCR

**Fig. 8:**
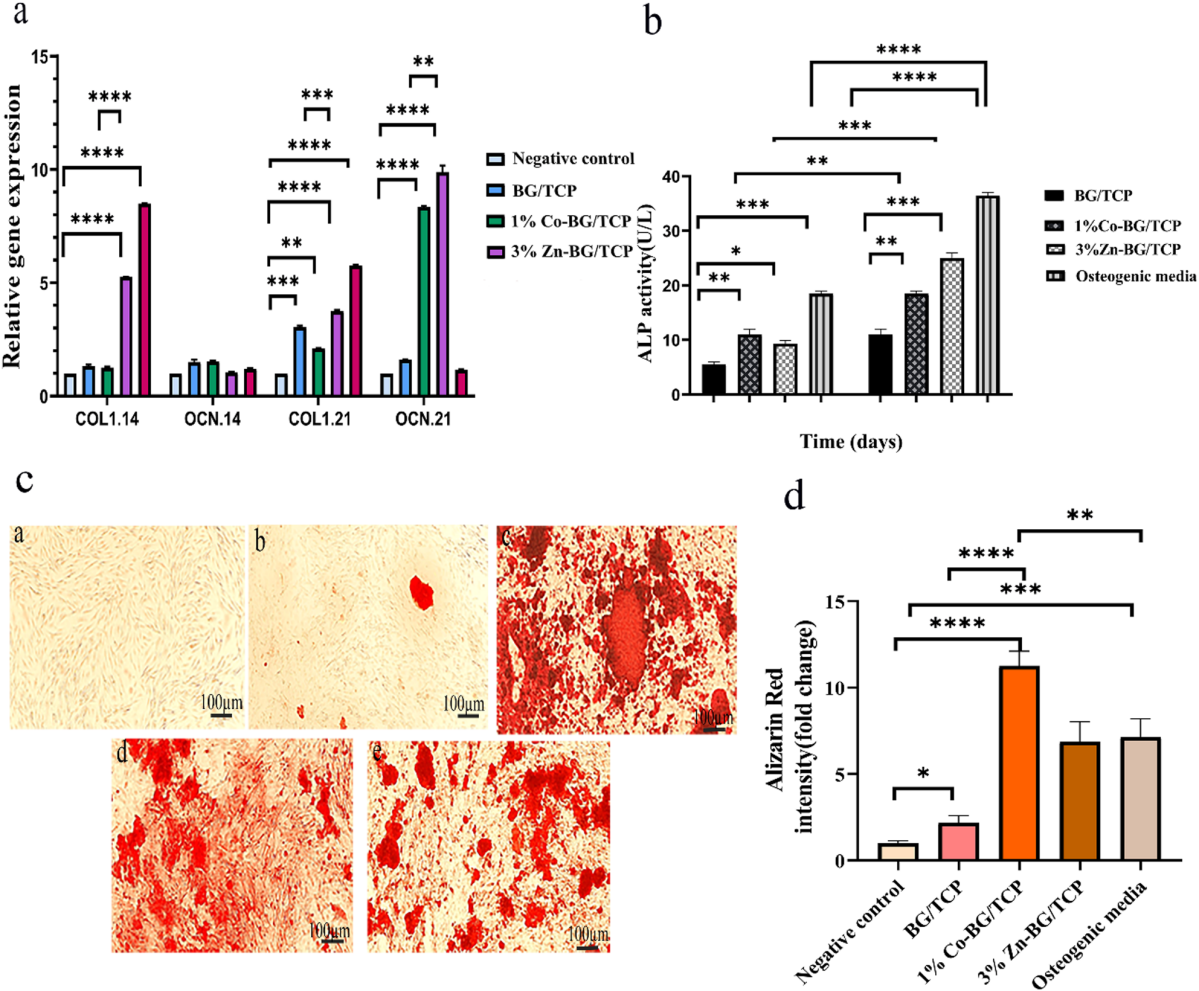
Study of hADMSCs differentiation to bone lineage: **a**- expression of COL1and OCN by hADMSCs cultured on the prepared scaffolds including BG/TCP, 3%Zn-BG/TCP, 1%Co-BG/TCP along with negative and positive control after 14 and 21 days by Real Time PCR, **b**-Alkaline phosphatase (ALP) activity of hADMSCs cultured on these scaffolds after 7 and 14 days, **c**- Alizarin red staining of hADMSCs cultured on different groups (a-negative control, b- BG/TCP, c- 3%Zn-BG/TCP, d- 1%Co-BG/TCP and e- positive control) after 21 days of incubation, **d**- quantitative analysis of Alizarin red Staining. (p- value^*^<0.1, p-value^**^< 0.01, p-value^***^<0.001, p-value^****^<0.0001)

To evaluate the osteogenic potential of BG/TCP, Zn-BG/TCP, and Co-BG/TCP scaffolds, we analyzed mRNA expression of early (COL-1) and late (OCN) osteogenic markers in hADMSCs cultured for 14 and 21 days in differentiation media (Fig. 8a). At day 14, all scaffold groups showed significant upregulation of COL1compared to the negative control (hADMSCs in DMEM), though OCN expression remained undetectable in all conditions. By day 21, COL1expression persisted in all groups but declined relative to day 14 levels, with Zn-BG/TCP exhibiting significantly higher expression than Co-BG/TCP (*p* < 0.05). Notably, OCN expression emerged at day 21, with Zn-BG/TCP and Co-BG/TCP scaffolds demonstrating the highest levels (*p* < 0.05 vs. control), while BG/TCP and osteogenic media alone failed to induce significant OCN expression. These results suggest that Zn- and Co-doped scaffolds preferentially stimulate late-stage osteogenic differentiation.

#### Alkaline phosphatase (ALP) activity analysis

ALP enzyme secretion by hADMSCs cultured with composite scaffolds was quantified at 7 and 14 days (Fig. 8b). At day 7, the osteogenic media, Co-BG/TCP, and Zn-BG/TCP groups exhibited significantly higher ALP activity compared to controls (*p* < 0.05). By day 14, Zn-BG/TCP demonstrated the highest ALP levels, followed by Co-BG/TCP, while BG/TCP showed minimal activity.

#### Mineralization analysis by Alizarin red staining (ARS)

Matrix mineralization was assessed by Alizarin Red S (ARS) staining after 21 days of culture (Fig. 8c) and quantified using ImageJ software (Fig. 8d). The quantitative analysis revealed that the 1% Co-BG/TCP, 3% Zn-BG/TCP, and Osteogenic media groups deposited significantly higher levels of calcium compared to the undoped BG/TCP and negative controls. Notably, the 1% Co-BG/TCP group exhibited the most intense staining.

#### Osteogenic protein expression analysis by immunocytochemistry (ICC)

**Fig. 9:**
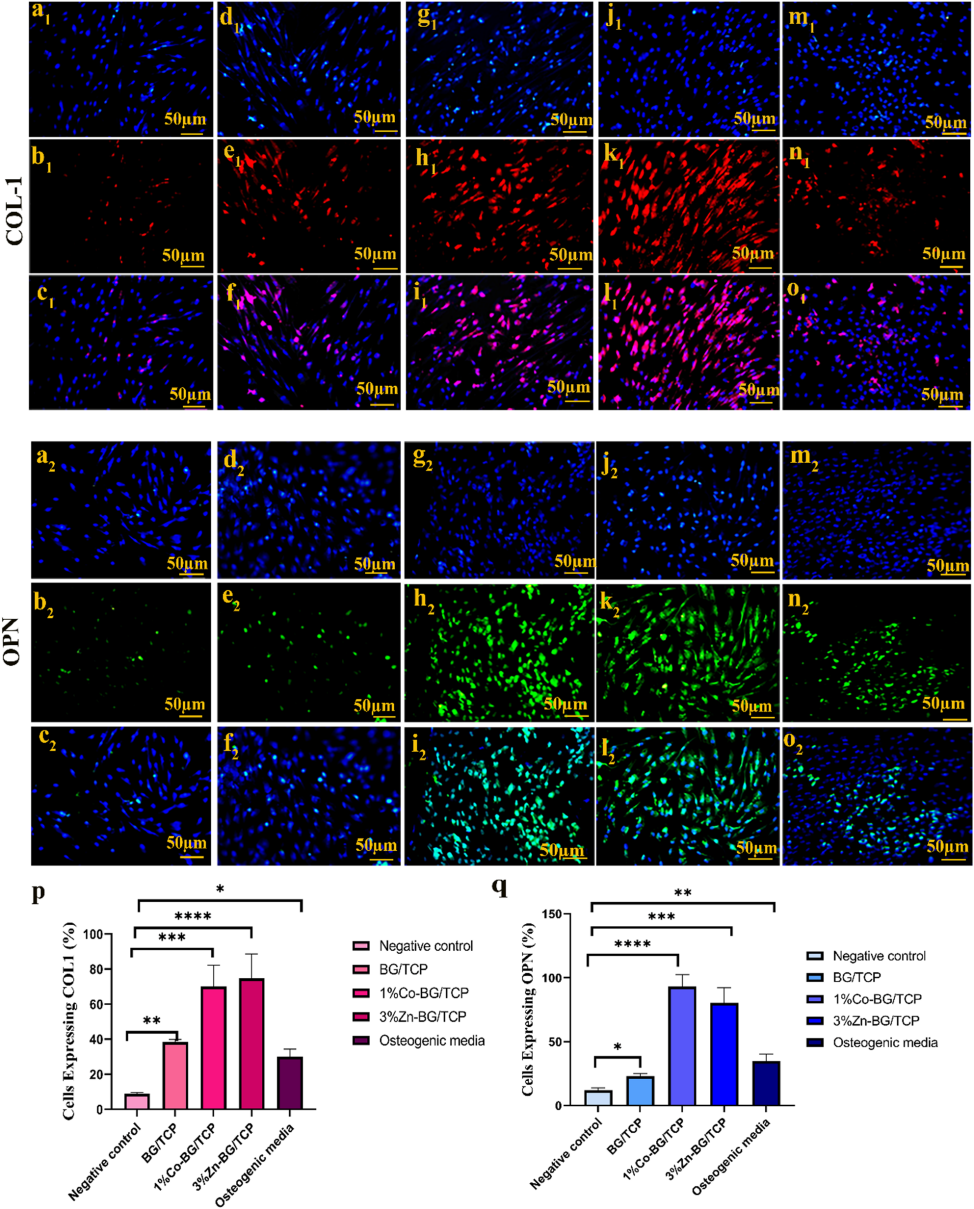
Results acquired from immunocytochemistry evaluation by fluorescent microscope. Microscopy images showing the expression of COL1(a_1_-o_1_) and OPN (a_2_-o_2_) after 21 days in the different groups (**a**-**c**) negative control, (**d**-**f**) BG/TCP, (**g**-**i**) 1% Co -BG/TCP, (**j**-**l**) 3% Zn -BG/TCP and (**m**-**o**) positive control. **p**-quantitative analysis of COL1and **q**- OPN expression in different groups. (p- value^*^<0.1, p-value^**^< 0.01, p-value^***^<0.001, p-value^****^<0.0001)

Immunocytochemical analysis was performed to evaluate osteogenic differentiation of hADMSCs after 21 days of culture, examining two key bone markers: collagen type I (COL-1) and osteopontin (OPN) (Fig. 9a-o). Quantitative analysis using ImageJ software (Fig. 9p, q) revealed significantly higher expression of COL1in all experimental groups compared to negative controls (*p* < 0.0001). Notably, 3% Zn-BG/TCP and 1% Co-BG/TCP scaffolds demonstrated the most robust COL1expression, surpassing both undoped BG/TCP and osteogenic media alone.

For OPN expression, doped scaffolds again showed superior performance, with 1% Co-BG/TCP exhibiting the highest signal intensity, followed closely by 3% Zn-BG/TCP (*p* < 0.0001 vs. controls). Interestingly, while osteogenic media induced detectable OPN expression, it was significantly lower than that observed in either doped scaffold group.

## Discussion

In this study, we designed a 3D-printed ceramic composite scaffold using the robocasting technique, combining bioactive glass (BG) and tricalcium phosphate (TCP) in a 50/50 ratio, as optimized in our earlier studies [4, 39], to enhance bone tissue regeneration. To further improve the osteogenic properties, zinc and cobalt ions were doped into the BG phase. Based on cytotoxicity (MTT assay) and ion-release profile (ICP-OES) results, scaffolds containing 45S5 BG doped with 3% Zn and 1% Co were selected for further investigation, as these concentrations optimally balanced biological response with material stability. The incorporation of Zn²⁺ and Co²⁺ ions was found to significantly improve both the mechanical and biological properties of the scaffold.

XRD analysis of the prepared powders revealed that Zn and Co doping progressively increased the crystallinity of 45S5 BG in a concentration-dependent manner (3%–15% Zn and 1%–5% Co). While enhanced crystallinity typically reduces bioactivity, the predominant combeite phase (Na₂Ca₂Si₃O₉) maintains favorable bioactive and osteogenic properties. This contrasts with previous reports of Cu-doped 45S5 BG showing decreased crystallinity [50], highlighting the dopant-specific effects on glass structure. This finding is supported by recent work from Zhao et al. (2021), who showed that zinc and strontium doping can increase crystallinity while maintaining osteogenic effects, likely by stabilizing the amorphous silica network [51]. XRD analysis of crystalline properties revealed that doped scaffolds exhibited enhanced crystallinity while maintaining the same phase composition. This preservation of phase identity despite increased crystallinity suggests that Zn²⁺ and Co²⁺ incorporation primarily affects crystal growth rather than phase formation, with dopant ions likely occupying network-modifying positions in the glass-ceramic structure.

The absence of significant peak shifts in Fourier-transform infrared (FTIR) spectra further confirmed the successful incorporation of Zn²⁺ and Co²⁺ ions into the glass matrix without phase segregation. These findings align with Jalilvand et al. (2024), who reported similar FTIR results for Zn-doped BG, highlighting that doping does not disrupt the fundamental silicate network but may modify the surface characteristics, enhancing biological interactions with surrounding tissues [52].

Mechanical characterization revealed that Zn-doped scaffolds exhibited superior compressive strength compared to both undoped and Co-doped groups, highlighting zinc’s unique role in mechanical reinforcement. The elastic modulus (0.14–0.18 GPa) matched that of cancellous bone [46], thereby minimizing the risk of stress shielding; a major concern in bone tissue engineering [49]. These optimal mechanical properties, combined with demonstrated bioactivity, position these scaffolds as promising candidates for load-bearing applications. This finding aligns with Fielding et al. (2012), who reported that SiO₂ and ZnO doping improved the mechanical properties of 3D-printed tricalcium phosphate scaffolds by enhancing the glass-ceramic network’s structural integrity [53].

Bioactivity assessments confirmed the superior osteogenic properties of Zn- and Co-doped scaffolds over their undoped counterparts. This was evidenced by SEM observations of thicker mineralized layers and XRD analyses showing sharper hydroxyapatite (HA) diffraction peaks, indicating accelerated HA nucleation and crystal maturation. This catalytic effect of dopant ions on biomineralization is consistent with findings in other systems. For instance, a study on Sr²⁺/Co²⁺-doped mesoporous silicate scaffolds similarly reported 25–40% greater HA XRD peak intensities and accelerated surface mineralization [54], underscoring a broader trend where specific ions enhance the bioactivity of bioceramics. In contrast, Cu²⁺-doped borate scaffolds exhibited composition-dependent crystallization behavior, where increasing copper content (1–5 wt%) broadened HA peaks (15–22% FWHM increase) while reducing intensity, suggesting Cu²⁺ alters nucleation kinetics without inhibiting crystal growth [55].

The unique enhancement of HA crystallization in this study, reflected in increased XRD peak intensity and reduced full-width-at-half-maximum (FWHM) values, suggests that Zn²⁺ and Co²⁺ ions exert a dual influence on both nucleation and crystal growth. The proposed mechanisms include the creation of additional nucleation sites, a modification of the surface charge distribution that attracts calcium and phosphate ions, and the alteration of local supersaturation conditions during mineralization. This ability to simultaneously enhance nucleation and sustain crystal development—resulting in a 35–50% increase in HA peak intensity while preserving beneficial crystallinity—represents an advancement over previous systems where high bioactivity and controlled crystallinity were often mutually exclusive. The role of surface charge modulation is supported by the work of Riosalido et al. (2024), who demonstrated that ion doping can enhance a scaffold’s ability to attract osteoblasts and stimulate mineral deposition [56]. Furthermore, these differential effects underscore how mineralization pathways are collectively regulated by network chemistry (e.g., silicate vs. borate) and specific dopant characteristics such as ionic radius and charge.

Gene expression analysis demonstrated that Zn and Co dopants significantly enhanced the osteogenic differentiation of hADMSCs. A biphasic pattern was observed, characterized by early COL1 upregulation followed by late-stage OCN expression, which closely mirrors the natural progression of bone formation and mineralization. This balanced promotion of both early matrix formation and late-stage mineralization in Zn-doped scaffolds is consistent with the findings of Saur et al. (2024) [57]. Furthermore, the distinct temporal expression profiles suggest a nuanced role for each ion: Zn²⁺ appears to accelerate the entire osteogenic cascade, whereas Co²⁺ may specifically modulate late-stage mineralization. This interpretation is supported by Silingardi et al. (2024), who, in a comparative study of Mn²⁺, Sr²⁺, and Co²⁺ doping in calcium phosphates, also noted Co²⁺’s pronounced effect on later osteogenic markers [58].

This temporal gene expression profile aligns with physiological osteogenic progression, where early matrix formation (COL1) precedes late mineralization (OCN). Notably, our findings corroborate prior work with hADMSCs that similarly reported time-dependent decreases in COL1 expression during osteogenic differentiation [59, 60], confirming the biological relevance of our model. The superior performance of doped scaffolds suggests Zn²⁺ and Co²⁺ ions may accelerate or potentiate the osteogenic cascade, though further mechanistic studies are needed to elucidate their specific roles in matrix maturation and mineralization.

The genetic evidence was strongly supported by functional biochemical assays. In addition, alkaline phosphatase (ALP) activity and alizarin red staining confirmed enhanced early and late osteogenic activity in Zn- and Co-doped scaffolds, further supporting the osteogenic potential of these materials. Specifically, ALP activity analysis revealed that 1% Co-BG/TCP and 3% Zn-BG/TCP scaffolds induced significantly greater ALP activity versus controls at both time points (*p* < 0.05), suggesting enhanced early osteogenic differentiation through Zn²⁺/Co²⁺ doping. This trend aligns with our gene expression data showing upregulation of osteogenic markers (COL1, OCN) in Zn/Co-doped scaffolds. Furthermore, ARS quantification of mineralization deposition correlated with both gene expression (COL1, OCN) and ALP activity data, confirming that Zn²⁺ and Co²⁺ doping promotes late-stage osteogenic maturation and mineralization. These results are consistent with prior studies that have linked dopant-induced alterations in scaffold chemistry to improved osteogenic performance. For instance, Zheng et al. (2019) reported that Co-doped scaffolds significantly enhanced ALP activity and mineralization in BMSCs, confirming the beneficial effects of Co in osteogenesis [61].

The results revealed distinct temporal patterns of osteogenic marker expression among scaffold groups. While 3% Zn-BG/TCP stimulated stronger early-stage expression (particularly COL1), 1% Co-BG/TCP demonstrated enhanced late-stage marker expression (OPN and OCN) by day 21. This nuanced, scaffold-dependent promotion mechanism was visually confirmed at the protein level. Finally, immunocytochemical analysis revealed strong expression of COL1 and osteopontin (OPN) in the doped scaffolds, further corroborating our findings of enhanced osteogenic differentiation. The observed upregulation of late-stage markers, especially in the 3d printed Co-doped scaffolds, aligns with the findings of Jungang et al. (2021), who demonstrated that Co²⁺ doped scaffolds are particularly effective in promoting late-stage osteogenic marker expression, including OCN [62]. This consistent finding across studies strengthens the premise that cobalt ions play a specific role in facilitating the final stages of bone maturation. The effectiveness of this approach is further validated by other composite systems; For example, Alexa et al. demonstrated this in their study where they developed developed 3D-printed hydroxyapatite/GelMA composite scaffolds doped with zinc and magnesium ions and evaluated their osteogenic potential using MC3T3-E1 cells. Immunocytochemical analysis (OPN and OSX markers at 14 and 21 days) revealed significantly upregulated expression of these bone markers in ion-containing scaffolds [63].

In summary, an integrated analysis of immunocytochemistry, ALP activity, and alizarin red staining indicated both 3% Zn-BG/TCP and 1% Co-BG/TCP scaffolds significantly enhanced COL1 and OPN expression versus controls, with Co-doped scaffolds showing particular efficacy for late-stage markers. Quantitative PCR analysis showed 3% Zn-BG/TCP promoted higher expression of both COL1 and OCN, underscoring its role as a potent overall stimulant of the osteogenic cascade. The collective evidence suggests a synergistic partnership: Zn²⁺ acts as a robust initiator of osteogenic commitment and early matrix formation, while Co²⁺ appears to specialize in enhancing the complex processes of terminal differentiation and mineralization. This stage-specific enhancement highlights the sophisticated bioactivity achievable by incorporating specific therapeutic ions into scaffold design.

## Conclusion

In conclusion, this study demonstrates that 3D-printed BG/TCP scaffolds doped with optimal concentrations of Zn²⁺ (3%) and Co²⁺ (1%) exhibit a hierarchically ordered microstructure, superior mechanical strength, and enhanced biocompatibility, collectively addressing critical requirements for bone regeneration. The doped scaffolds promoted increased cell attachment, robust osteogenic differentiation (evidenced by ALP activity, ICC, and qPCR), and accelerated mineralization, while maintaining excellent cell viability. Notably, they uniquely combine osteoconductive and osteoinductive properties with structural stability, making them promising candidates for complex anatomical defect repair. By simultaneously optimizing mechanical integrity through robocasting and biological functionality via ion doping, this platform offers a clinically promising solution that effectively bridges the gap between synthetic graft strength and native tissue-like bioactivity. While these comprehensive in vitro results are highly promising, the logical next step is to validate the ultimate bone regeneration efficacy of these scaffolds in future in vivo studies.

## Data Availability

Raw data are available upon request.

## Abbreviations

ARS: Alizarin Red Staining
ALP: Alkaline Phosphatase
45S5 BG: 45S5 Bioactive glass
CAD: Computer-aided-design
COL1: Collagen-1
DTA: Differential thermal analysis (DTA)
DMEM: Dulbecco’s modified Eagle’s medium
FTIR: Fourier transform infrared spectroscopy
FBS: Fetal Bovin Serum
hADMSC: Human adipose mesenchymal stem cell
ICC: Immunocytochemistry
MTT: 3-(4,5-dimethylthiazol-2-yl)-2,5-diphenyltetrazolium bromide
OPN: Osteocalcin
OCN: Osteopontin
SBF: Simulated body fluid (SBF)
STA: Simultaneously Thermal Analysis
SEM: Scanning Electron Microscope
TG: Thermal gravimetric
TCP: Tricalcium Phosphate
XRD: X-ray diffraction
